# The distribution and use of feedback bulletins among national immunization program management teams in East and Southern Africa

**DOI:** 10.11604/pamj.supp.2020.35.1.19062

**Published:** 2020-01-03

**Authors:** Balcha Girma Masresha, Goitom Weldegebriel, Emmaculate Lebo, Jethro Chakauya, Daniel Fussum

**Affiliations:** 1 WHO Regional Office for Africa, Brazzaville, Congo; 2 WHO Inter-country Team for Eastern and Southern Africa, Harare, Zimbabwe

**Keywords:** Immunization, monitoring, feedback

## Abstract

**Introduction:**

Immunization program monitoring includes numerous activities, some of which include monitoring of vaccination coverage, surveillance performance and epidemiological patterns. The provision of timely, high quality and actionable feedback is an essential component of strengthening health systems. Within the African region of the WHO, various bulletins are produced and disseminated regularly to provide feedback on the performance of immunization programs and vaccine preventable disease control initiatives.

**Methods:**

The 2019 annual national immunization program managers’ meeting for countries in the eastern and southern African subregion was held in Asmara from 18 - 20 March 2019. A survey questionnaire was administered to the participants representing the national programs and in-country partners across the 20 countries.

**Results:**

On average, the 75 respondents receive 1.8 e-mailed feedback bulletins monthly. Twenty-three (31%) respondents receive 3 or more written feedback bulletins per month, and 72% receive the bulletins regularly. On a scale of 1 - 5 (from lowest to highest), 87% participants rated the relevance of the bulletins they receive at 4 - 5. Only 19% of the respondents responded that the results are discussed within the national immunization program, and 14% stated that action points are generated based on the feedback received. Fifty-nine (79%) respondents want to receive more frequent feedback on routine immunization performance. Among the EPI program managers and the EPI program data managers, the access to these feedback bulletins was quite limited. Even though the primary objective of the bulletins is to initiate discussions and action based on the provided feedback, such discussions do not happen regularly at country level. The programmatic use and advocacy value of the bulletins is not optimal.

**Conclusion:**

We recommend integrating program feedback, regularly updating the distribution lists, the additional use of instant messaging platforms for distribution, as well as online posting of the bulletins for wider availability.

## Introduction

The African Regional Strategic Plan for Immunization 2014 - 2020 (RSPI) maps out ambitious goals for improving access to vaccines and to eliminate targeted vaccine preventable diseases. The plan acknowledges the need to position strong immunization systems as an integral part of well-functioning health systems, and recommends corresponding actions for countries, one of which is to enhance the collection, triangulation and use of administrative, surveillance, risk assessment and vaccine safety data to improve performance of immunization services and complementary actions in tackling the disease burden [1]. Immunization program monitoring is done regularly by recording and tracking service data, including the number of doses of antigens provided to persons in the target population. Immunization coverage data needs to be interpreted alongside information from vaccine preventable disease surveillance systems in order to provide a more complete understanding of the performance and impact of immunization programs in the control of vaccine preventable diseases. The monitoring and use of data for action is one of the five pillars of the “Reaching Every District” approach used to address common obstacles to increasing immunization coverage [2]. At the district level, regular review of program and health worker performance has been recognized as one of the key drivers of improvement of routine immunization systems in the African setting [3]. The development of feedback mechanisms that facilitate access to timely, feasible, cost-effective and actionable performance data is an essential component of strengthening health systems. Feedback mechanisms in health systems provide opportunities for learning, and help build accountability into the system [4]. The provision of feedback is essential to motivate health workers, assure adherence to standards, track progress towards national and regional goals, provides corrective actions and to align and prioritize technical support. Feedback may be provided using various approaches including during supervisory visits, periodic program performance review meetings, using written bulletins, among others [3–6]. In disease surveillance systems, feedback is considered one of the core activities [7–9]. One of the core functions of the World Health Organization includes monitoring the health situation and assessing health trends [10]. In the area of immunization and the control vaccine preventable diseases, countries regularly report coverage and disease incidence data through the WHO country and Regional offices. One important example of monitoring from the WHO global level is the compilation of national reported data through the WHO-UNICEF joint reporting form, and subsequent generation of antigen-specific annual estimates of coverage for each country, often referred to as the WHO-UNICEF estimates of national coverage [11].

Using programmatic data generated at the national level, the WHO African Regional immunization and polio eradication programs provide regional programmatic overview and feedback to national immunization programs during annual meetings, and periodic monitoring and evaluation exercises, in the form of written program summaries, presentations and reports. In addition, regular feedback bulletins are produced to monitor country progress against the targets and to present a comparison of performance between different countries. The primary aim of these bulletins is to provide a regular and transparent assessment of country performance, with a view to encourage progress, and indicate the need for course correction where needed. Within the African region of the WHO, the provision of written surveillance feedback is a recognized legacy of the polio eradication program [12–14]. Various feedback bulletins are produced and disseminated regularly highlighting information on routine immunization performance and vaccine preventable disease control initiatives. The emailed feedback bulletins destined to reach the countries in the East and southern subregion include: Regional: a) Weekly African Regional polio updates; b) Weekly Regional polio lab feedback tables; c) Monthly African Regional immunization bulletin; d) Monthly Regional measles-rubella surveillance feedback summary bulletin. Sub-regional: a) Polio Surveillance Weekly Updates for East and Southern Africa; b) Monthly integrated EPI feedback bulletin for East and Southern Africa subregion; c) Monthly sub-regional Integrated Supportive Supervision Feedback; d) Quarterly sub-regional feedback bulletin on Rotavirus and Pediatric Bacterial Meningitis sentinel surveillance. Even though efforts are made to ensure the relevance of these feedback products, not much is known with regards to exactly how this information is received and utilized at country level. This study attempts to shed light on the utility of the written programmatic feedback in countries in eastern and southern Africa.

## Methods

National immunization and disease control programs regularly share immunization coverage and disease surveillance databases with WHO. Country program data on acute flaccid paralysis (AFP) surveillance, polio virology laboratory as well as measles case-based surveillance and serological lab data is shared weekly, while other vaccine preventable disease (VPD) surveillance databases (eg., neonatal tetanus surveillance, meningitis surveillance, sentinel surveillance for pediatric bacterial meningitis and severe childhood diarrhea) and immunization coverage monitoring data is shared monthly. The immunization program data management team at the sub-regional office of the WHO aggregates, cleans and analyses the data regularly with the relevant program officers in order to provide interpretation, prepare and disseminate feedback bulletins. These bulletins include tabulation, maps and charts as well as narrative descriptions covering data quality, immunization coverage, surveillance and laboratory performance, as well as epidemiological trends.

The WHO African regional immunization program develops and disseminates more detailed and specialized feedback bulletins summarizing Regional performance across the 47 countries. These comprise of the weekly feedback from the polio program on the Acute Flaccid Paralysis surveillance performance and Polio Laboratory performance, monthly feedback on the measles and rubella surveillance performance, and the monthly routine immunization newsletters. While there may be some differences in the target audience of these feedback bulletins, the national immunization and disease surveillance program staff remain at the primary targets. Every year, the WHO and UNICEF Regional offices jointly organize a meeting of national immunization program managers, to share programmatic information and experiences, monitor performance against regional and global targets and goals and discuss scientific updates. These annual sub-regional level meetings are also attended by global and regional partners. The 2019 annual national immunization program managers’ meeting for 20 countries in the Eastern and southern African subregion was held in Asmara, Eritrea from 18 - 20 March 2019. The participants included immunization program managers, data managers, other national program team members, as well as national level partners from the 20 countries. A survey questionnaire was administered to the participants representing the national program and in-country partners across the 20 countries. The questionnaire focused on the programmatic feedback provided to countries from the WHO regional and sub-regional levels. The data was entered and analyzed using MS Excel.

## Results

The questionnaire was distributed to 91 persons, and responses were received from 76 participants. One questionnaire was discarded because of incompleteness. Participants from all 20 countries in the subregion provided responses to the survey questions, with at least 2 respondents from each country in the subregion except Mozambique, which had only one response submitted. The 41 participants from various in-county partners were from WHO, UNICEF, John Snow Inc. (JSI), Clinton Health Access Initiative (CHAI), PATH and Aspen Management Partnership for Health (AMP Health). A third of the respondents had 10 years or more of experience with the immunization work at the national level. On average, the respondents receive 1.8 e-mailed feedback bulletins at least monthly. Twenty-three (31%) respondents receive 3 or more written feedback bulletins per month (Table 1). The 15 national immunization program managers or directors who responded to the survey indicated that they receive on average 1.8 feedback bulletins over the course of a month. On the other hand, 9 of the 18 national immunization program officers responsible for immunization data management (or monitoring and evaluation) received no more than one feedback bulletin per month.

**Table 1:** Characteristics of respondents and feedback bulletins received

	Ministries of Health	In-country partner agencies
Number of respondents	34	41
Average years of experience at national level	6.8 years	9.8 years
Average number of feedback bulletins received at least monthly	1.5	2.2

The majority (72%) of the respondents receive the feedback bulletins quite regularly, while 18 get them irregularly. On a scale of 1 - 5 (from lowest to highest), 47 of 54 (87%) participants rated the relevance of the bulletins they receive at 4 - 5, while 42 (91%) of 46 respondents stated that the feedback bulletins were detailed enough in their content and rated them 4 - 5. With regards to how the feedback bulletins are received at the country level, only 19% responded that the results are discussed within the national immunization program, and 14% stated that action points are generated based on the feedback received (Table 2). In the future, 49 (65%) would like to see more detailed feedback and content on routine immunization coverage performance, 47 (63%) on data quality, while 31 (41%) would like to see more information on VPD outbreaks in the subregion. With regards to the frequency of feedback, 59 (79%) respondents want to receive more frequent feedback on routine immunisation performance, while 33 (44%) wanted more feedback on surveillance of rotavirus and pediatric bacterial meningitis (surveillance of diseases targeted by the newer vaccines), 48 (64%) on measles and rubella elimination, 38 (51%) on polio eradication and 12 (16%) on maternal and neonatal tetanus elimination.

**Table 2:** Use of feedback bulletin for program action at country level

	Results in feedback bulletins discussed at national level	National level generates action points based on feedback received	Feedback bulletins shared with decision makers	Technical support from WHO aligned with performance issues as indicated in the feedback bulletins
Never	13 (19%)	9 (14%)	7 (12%)	1 (2%)
Sometimes/rarely	37 (55%)	35 (56%)	38 (67%)	39 (65%)
At least once a month	17 (25%)	19 (30%)	12 (21%)	20 (33%)
**Total responses**	**67 (100%)**	**63 (100%)**	**57 (100%)**	**60 (100%)**

## Discussion

A major objective of providing written feedback to national officers, tabulating and mapping performance across multiple countries, is to allow immunization program managers at subnational and national levels to see their work within the bigger context of the Regional and global goals. In this regard, it is critical that they get accurate, timely, relevant feedback that also provides programmatic guidance and is followed up with the appropriate technical assistance. Currently, all of the immunization program feedback bulletins from the WHO African regional and sub-regional levels that are provided to the national level are shared by e-mail, and none of these bulletins are posted online, which limits the audience of the bulletins. The participants in this survey are the technical leaders and partners for immunization and VPD control work in their respective countries. Given that most of the respondents have been responsible for immunization activities for a number of years, it is expected that they are already familiar with the feedback processes and products. The majority of the respondents rated the feedback bulletins they receive as relevant and detailed enough. Despite this, our study has highlighted needs for improvement in the distribution and utilization of the feedback bulletins. Even among the EPI program managers and the EPI program data managers, the access to these feedback bulletins was quite limited. Only a third of the total respondents receive three or more feedback bulletins a month. The primary objective of the feedback is to monitor performance across multiple countries, with a view to initiate discussions and action as necessary. However, only 17 (23%) responded that such discussions happen regularly at country level. Only 21% of the participants responded that these bulletins are brought to the attention of higher-level decision makers, limiting the advocacy value of the bulletins.

Periodically, countries are supported to do immunization program and/or surveillance reviews, and other similar in-depth program assessment activities to identify their strengths and weaknesses, and address gaps that hinder the attainment of program objectives. However, such exercises are resource intensive, conducted once every 3 - 5 years and cannot replace the frequent provision of program feedback. In many countries, the national immunization program and the surveillance / disease control program are in separate divisions within ministries of health. In such cases, the responsibility for VPD surveillance exists in a program outside of the immunization program. It is expected that these two programs work closely in terms of planning interventions, data sharing and impact monitoring among others. However, multiple national program reviews have identified gaps in information sharing and use in such contexts [15]. In the past two decades, countries have introduced new and under-utilized vaccines, and recently introduced a life-course approach to immunization beyond infancy. With this comes increased complexity of vaccination schedules, increased expectations with regards to monitoring data quality, as well as the need for continuous capacity building to refine technical and managerial skills at all levels [1, 16]. Within such a rapidly evolving and dynamic context, the provision of high quality program feedback is critical to improve overall program management capacity and data quality. Robust monitoring and accountability frameworks are a critical part of improving immunization programs [17]. In addition, the generation of feedback helps improve the immunization monitoring system itself by identifying and highlighting issues related to the monitoring process and data quality [18].

With the adoption of Demographic Health Information Systems (DHIS2), countries are preparing to move into web-based real-time data entry and data management platforms that provide the functionality of generating automated dashboards as well as alerts and reports. However, the linkage of data outputs with programmatic guidance will continue to be relevant and will not replace the need for high quality program feedback [19]. This is the first such study to attempt to provide an insight into the perceptions towards, the distribution and utilization of programmatic feedback within the regional immunization programs. However, this study is limited in scope to the 20 countries and specifically to the participants of the annual immunization program managers’ meeting for the East and southern Africa subregion. The meeting participants representing the national ministries of health were mostly from the respective national immunization programs and not necessarily technical staff from the national disease surveillance and/or disease control units responsible for handling VPD surveillance. The study did not also attempt to delve into the contents and format of each feedback bulletin.

## Conclusion

Written feedback is a critical element for strengthening public health programs. The written feedback provided by the WHO on the immunization and vaccine preventable disease efforts in the subregion can be improved through the use of updated distribution lists, the additional use of instant messaging platforms for distribution, as well as online posting of program feedback bulletins for wider and longer periods of availability. In addition, bulletins should be better integrated and regularly shared with the inclusion of programmatic guidance to better guide countries towards the RSPI targets. National programs should create regular platforms to review performance widely across the immunization and surveillance programs, and explore ways of utilizing the feedback to improve data quality and overall program performance.

### What is known about this topic

- Monitoring and use of data for action is one of the five pillars of the “Reaching Every District” approach;
- The provision of timely, high quality and actionable feedback is an essential component of strengthening health systems;
- The WHO Regional and Sub-regional levels share various emailed program feedback bulletins covering immunization and vaccine preventable disease control initiatives regularly.

### What this study adds

- The various feedback bulletins from the WHO regional and sub-regional levels are not reaching all the key program staff at country level;
- All the national immunization programs are not regularly discussing the feedback results and generating action points based on the findings;
- There is a need to explore different approaches to widely sharing the feedback, and making it more useful and actionable for countries.

## Competing interests

The authors declare no competing interests.
